# Updates in endocrine therapy for metastatic breast cancer

**DOI:** 10.20892/j.issn.2095-3941.2021.0255

**Published:** 2021-10-05

**Authors:** Poorni M. Manohar, Nancy E. Davidson

**Affiliations:** 1 University of Washington/Seattle Cancer Care Alliance, Seattle 98109, WA, USA; 2 Fred Hutchinson Cancer Research Center, Seattle 98109, WA, USA

**Keywords:** Endocrine therapy, metastatic breast cancer

## Abstract

Endocrine therapy (ET) remains the mainstay of treatment for steroid hormone receptor-positive, human epidermal growth factor 2 (HER2)-negative metastatic breast cancer (MBC). Tumor resistance to hormone therapy has led to the development of novel endocrine drug combinations, transforming the landscape of MBC management. The options for ET are expanding, with promising agents in the pipeline. Although MBC remains incurable, many patients can enjoy years of survival with good quality of life by cycling through the many available agents. With the plethora of available agents and rapid approvals, clinicians look to evidence-based guidelines to assist in treatment selection to maximize patient well-being. In this review, we provide a contemporary review of the advances in ET and a suggested algorithm to guide clinicians in daily management of patients with hormone receptor-positive, HER2-negative MBC. We will discuss landmark trials and highlight their impact in reshaping treatment approaches. Finally, we will provide a glimpse into advances on the horizon and the promise they bring to improve outcomes in patients with advanced breast cancer.

## Introduction

Endocrine therapy (ET) is the foundation of treatment for patients with breast cancer driven by expression of estrogen receptor (ER) and/or progesterone receptor (PR)^1^. This practice dates back to the late 19th century when George Beatson reported that the removal of ovaries improved outcomes for young women with advanced breast cancer^2^. More than half a century later, the ER was discovered, shedding light on the role of estrogen in breast cancer^3^. Today, research has advanced our understanding of advanced breast cancer pathophysiology, leading to identification of new targets to complement ET such as mammalian target of rapamycin (mTOR) inhibitors, cyclin-dependent kinase 4/6 (CDK4/6) inhibitors, histone deacetylase (HDAC) inhibitors, and phosphoinositide 3 kinase inhibitors.

The rapid development and approvals of new therapies can pose clinical challenges to the identification of the best treatment selection for patients. Here we provide a suggested framework for treatment of patients with hormone receptor-positive metastatic breast cancer (MBC) and highlight novel agents and approaches to bolster evidence-based clinical practice. Treatment of metastatic ER- and PR-negative or human epidermal growth receptor 2 (HER2)-positive breast cancer follows different algorithms and is not within the scope of this review. In our review, the term “hormone receptor” denotes “ER and/or PR”.

## Methods

We searched PubMed for English-language articles related to the treatment of MBC, with a focus on patients with ER-positive disease. We narrowed our search to emphasize large randomized clinical trials, meta-analyses, updates from national meetings, and guidelines from major professional societies. A comprehensive review was performed for articles published from January 1, 2000 to November 23, 2020. Articles agreed on by both authors to define modern clinical practice and represent recent research advances in MBC were included.

## Principles of therapy: MBC

**Figure 1:**
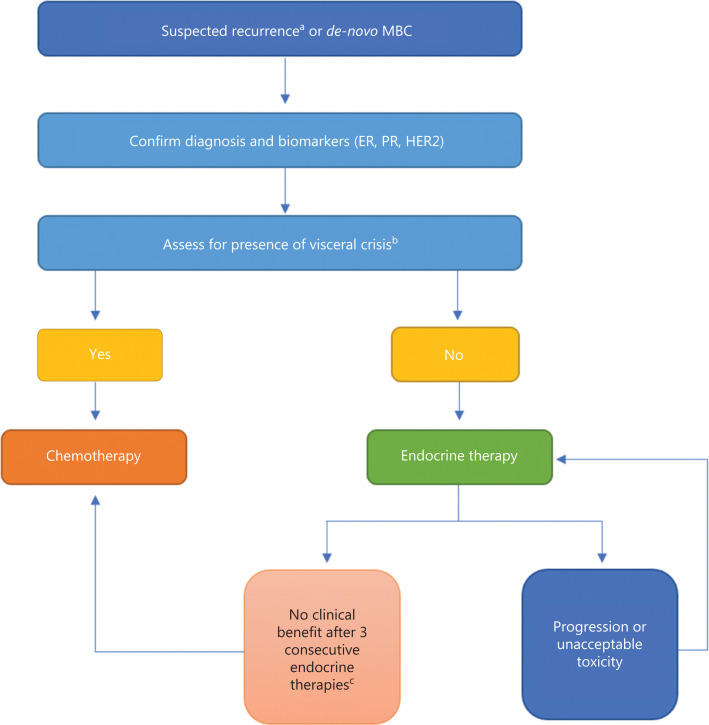
Treatment approach to patients with metastatic hormone receptor-positive, HER2-negative MBC. ^a^Patients with diagnosis of MBC during endocrine therapy or within 1 year of endocrine therapy are a select population and discussed separately. ^b^Definitions of visceral crisis vary, but significant threat to organ function by burden or location of metastases can be considered a visceral crisis. ^c^Tumor testing for actionable mutation such as phosphatidylinositol-4,5-bisphosphate 3-kinase catalytic subunit alpha should be pursued prior to chemotherapy selection unless visceral crisis develops. HER2, human epidermal growth factor 2; MBC, metastatic breast cancer; ER, estrogen receptor; PR, progesterone receptor.

Approximately 2.2 million new cases of breast cancer were diagnosed worldwide in 2020, with 5%–10% diagnosed at metastatic stage and 20%–30% predicted to recur with metastases^4–6^. Because MBC is treatable but not curable, clinicians should engage in shared decision making with patients to focus on maximizing quality and quantity of life^7^. **Figure 1** summarizes the general approach to treatment selection.

For patients with MBC, establishing hormone receptor status at diagnosis and recurrence is a critical determinant of treatment selection and disease course^8,9^. Discrepant hormone receptor expression between the primary tumor and metastatic site is seen in up to 20% breast cancer patients, emphasizing the need for confirmation of hormone receptor status^10,11^. Thus, most guidelines strongly recommend pathological confirmation of metastatic disease before initiation of first-line therapy in metastatic disease^8,9^.

The next key step in the selection of therapy requires assessment of patient symptoms and burden of disease. Typically, patients with hormone receptor-positive cancer are treated with ET, and chemotherapy is reserved for subsequent lines of treatment. However, for patients with a large burden of disease who appear to be in visceral crisis, front-line chemotherapy may be necessary to achieve a quick response^12^. There is no uniformly accepted definition for visceral crisis; in general, a significant threat to organ function because of disease burden or location of metastases can be considered a visceral crisis. Presence of lymphangitic lung metastases, bone marrow replacement, carcinomatous meningitis, or significant liver metastases would fall into this category^9^. The final consideration prior to selection of treatment is the time interval between receipt of ET and relapse. Typically, patients who develop metastatic disease during or within 1 year of completing adjuvant ET should be given careful consideration before initiating ET^8,9^. These patients may demonstrate endocrine resistance and require alternative approaches in initial treatment to re-sensitize the tumor to ET. However, emerging evidence suggests that ET may still be beneficial in this population^13^.

**Figure 2:**
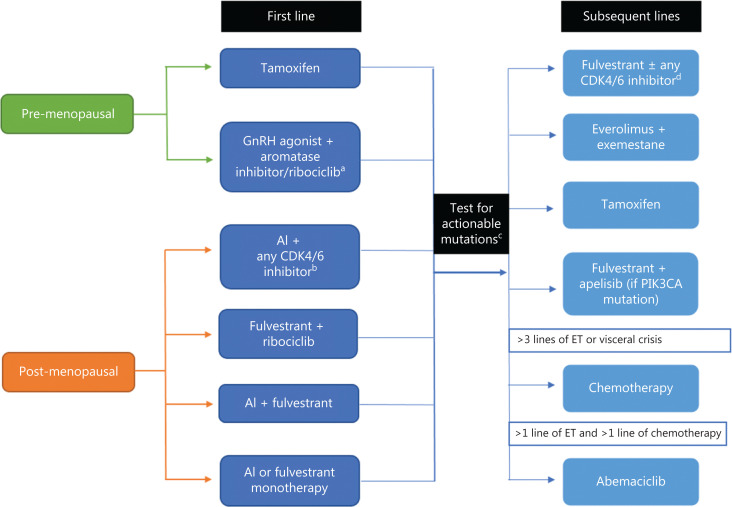
Simplified approach to management of hormone receptor-positive, HER2-negative MBC. ^a^Palbociclib or ribociclib can be used. ^b^Three currently approved agents in the United States include palbociclib, ribociclib, and abemaciclib. ^c^After first-line therapy, consider testing for actionable mutations *via* next-generation sequencing of tumor or circulating tumor DNA, specifically to assess for PIK3CA mutation. ^d^Endocrine therapy in combination with CDK4/6 inhibitors can be used in subsequent lines for patients who have not received CKD4/6 inhibitor therapy. HER2, human epidermal growth factor 2; MBC, metastatic breast cancer; CDK4/6, cyclin-dependent kinase 4/6; GnRH, gonadotropin-releasing hormone; AI, aromatase inhibitor; PIK3CA, phosphatidylinositol-4,5-bisphosphate 3-kinase catalytic subunit alpha.

The re-assessment of hormone receptor status, evaluation for visceral crisis, and understanding of mechanisms of endocrine resistance arm clinicians with the knowledge to identify the best strategy for first-line treatment. The next step is to select the appropriate monotherapy or combination therapy to provide the best outcome for the patient. This task can be daunting with the myriad of available and preferred first-line options^8^. The purpose of this review is to provide a simplified, comprehensive, and evidence-based approach to treat newly diagnosed patients with hormone receptor-positive, HER2-negative MBC (**Figure 2**). Importantly, there are no data on whether combining ET (with or without targeted agents) with chemotherapy improves overall survival (OS), and therefore, we do not recommend this strategy^14^.

## Selection of ET

For patients without visceral crisis, ET remains the backbone of therapy for hormone receptor-positive, HER2-negative MBC. Historically, endocrine monotherapy aimed at either depleting the estrogen ligand [aromatase inhibitors (AIs) in postmenopausal women or ovarian function suppression in premenopausal women] or targeting ER signaling pathways (selective ER modulators or deregulators) has been the standard of care. Many studies have compared outcomes in post-menopausal women treated with AI *vs.* tamoxifen in MBC^15–17^. In aggregate, they demonstrated that AIs improved progression-free survival (PFS) compared with tamoxifen, but no OS benefit was noted. The studies also suggested that all 3 AIs are essentially equivalent and interchangeable as first-line options. However, acquired resistance to hormonal blockade launched the search for new strategies and resulted in the discovery of novel combination therapies^18^.

Although combination ET has not been generally shown to improve outcomes over sequential monotherapy, investigators hypothesized an ER down-regulator (fulvestrant) may have increased effectiveness compared with AI (anastrozole)^19^. The FALCON trial showed a significant PFS benefit with fulvestrant [16.6 *vs.* 13.8 months; hazard ratio (HR) 0.797; 95% CI, 0.64 to 0.99; *P* = 0.04] compared with AI therapy alone in the first-line setting. The effect was most apparent in patients who had not received adjuvant ET, suggesting that patients with *de novo* or hormone-treatment naïve MBC may derive the most benefit. A similar finding was noted in the SWOG0226 phase III trial where addition of fulvestrant to NSAI was compared with fulvestrant alone in *de novo* hormone receptor-positive MBC^20^. The combination therapy had a longer OS of 49.8 months compared with 42 months with fulvestrant alone (HR 0.82; 95% CI 0.69 to 0.98; *P* = 0.03).

## Combination therapy: PIK3CA, mTOR inhibitors, and HDAC inhibitors

Next-generation sequencing of tumors catalyzed a movement to search for actionable mutations. It is estimated that approximately 40% of patients with hormone receptor-positive, HER2-negative MBC may harbor activating mutation(s) in the phosphatidylinositol-4,5-bisphosphate 3-kinase catalytic subunit alpha (PIK3CA)^21^. The SOLAR-1 study showed that the combination of a PIK3CA inhibitor (alpelisib) with fulvestrant improved PFS by 9 months (20 *vs.* 11 months) compared with fulvestrant alone^22^. Patients included in this study received prior ET in the metastatic setting or had received an AI in the context of neoadjuvant or adjuvant therapy for advanced disease. An important caveat in the initiation of alpelisib is the need to monitor for hyperglycemia as 36% of patients experienced this grade 3/4 adverse event in the trials. Testing for PIK3CA mutation is typically done on tumor tissue, preferably a recent site of metastases to ensure accurate results. If tumor tissue is not available, plasma specimens and liquid biopsy may also assess PIK3CA mutations. However, this method may not be widely available, and its validity is still being investigated.

Activation of the mTOR intracellular signaling pathway has also been proposed as a mechanism of resistance to ET in MBC^23^. In patients who have progressed on first-line ET and remain without visceral crisis, second line therapy with ET and the mTOR inhibitor, everolimus, is an evidence-based option^24^. Everolimus plus exemestane was evaluated *vs.* exemestane in the BOLERO-2 study with the final analysis after 18 months of median follow-up demonstrating an significant PFS improvement with the combination (7.8 *vs.* 3.2 months; HR 0.45; 95% CI 0.38 to 0.54; *P* < 0.0001) compared with AI alone^25^. The combination was associated with more grade 3 and 4 anemia (6% *vs.* 1%), hyperglycemia (4% *vs.* 1%), and pneumonitis (3% *vs.* 0%), necessitating careful monitoring and assessment for symptoms.

Another proposed mechanism of resistance involves changes in gene expression secondary to epigenetic modifications, which might be reversed with the use of HDAC inhibitors^26^. The ENCORE 301 phase II randomized study demonstrated a significant improvement PFS and OS with the addition of HDAC inhibitor entinostat to exemestane in patients with hormone receptor-positive MBC with disease progression after prior NSAI^27^. These results prompted the development of E2112, a phase III registration trial which investigated entinostat or placebo in combination with exemestane in patients with locally advanced or MBC who have experienced disease progression after a NSAI. The final results of the trial presented at the 2020 San Antonio Breast Cancer Symposium (SABCS) did not show a benefit with the addition of entinostat^28^. The discordance between the phase II and phase III results are still being investigated, but it is possible that different eligibility criteria between the studies could explain the different findings. The study also emphasizes the need for confirmatory phase III data before clinical implementation of therapies from promising phase II results.

## Impact of CDK4/6 inhibitors

In the era preceding CDK4/6 inhibitors, the median survival of patients with MBC that is hormone-receptor positive, human epidermal growth factor receptor 2 (HER2)-negative ranged between 16 and 26 months^29^. The emergence of CDK4/6 inhibitors has noticeably shifted the landscape of MBC, leading to unparalleled improvement in PFS and OS^30–34^.

### Role of CDK4/6 inhibitors in first-line therapy

Preclinical studies showed that persistent cyclin D1 expression and constitutive activation of CDK4/6 are associated with endocrine resistance^35,36^. These data provided strong rationale to study CDK4/6 inhibitors in combination with hormone therapy. The benefit of CDK4/6 inhibitors to act in synergy with ET was first shown to be an effective first-line treatment option in a randomized, phase II clinical trial, PALOMA-1^37^. The findings were later validated in a phase III trial, PALOMA-2, where 666 post-menopausal women were randomized to receive letrozole monotherapy or letrozole with the CDK4/6 inhibitor, palbociclib^30^. A remarkable and statistically significant ~10-month PFS improvement was observed with the addition of palbociclib (24.8 *vs.* 14.5 months; hormone receptor 0.58; 95% CI 0.46 to 0.72; *P* < 0.001). There were more adverse events with the combination, including neutropenia (79.5%), fatigue (37.5%), nausea (35.1%), arthralgias (33.3%), and alopecia (32.9%). Although grade 3 neutropenia was noted in 56.1% of patients, this did not translate to clinically significant complications, as evidenced by the very low rates of febrile neutropenia^30^. Furthermore, subsequent meta-analyses with a focus on adverse events have confirmed that febrile neutropenia and related infections are low (1% and 3%, respectively) in patients receiving CDK4/6 inhibitors^38^. Patient reported outcomes also suggest that episodes of neutropenia are not associated with decreased quality of life^39^.

The landmark PALOMA-2 trial paved the way for further studies evaluating CDK4/6 inhibitors in MBC. Two other CDK4/6 inhibitors, ribociclib and abemaciclib, were studied and demonstrated similar outcomes, suggesting a class effect for efficacy. The MONALEESA-2 trial showed that the addition of ribociclib to letrozole significantly improved PFS over letrozole in post-menopausal patients treated in the first-line setting for hormone receptor-positive HER2-negative MBC^34^. Ribociclib carries a unique side-effect profile and requires monitoring of hepatic transaminases and an electrocardiogram as part of clinical management. Rare but serious complications of hepatotoxicity and QT prolongation have been reported in clinical trials^38^.

The MONARCH-3 trial placed abemaciclib on the map for first-line treatment of hormone receptor-positive MBC. This randomized phase III study evaluated abemaciclib in combination with a non-steroidal aromatase inhibitor (NSAI) in 493 post-menopausal women. At the final analysis at a median follow-up of 26 months, abemaciclib plus NSAI showed a significant PFS improvement of 28.1 months compared with 14.7 months for the NSAI-alone arm (HR 0.53; 95% confidence interval, CI 0.42 to 0.70; *P* = 0.000002)^40^. Diarrhea was the leading cause of toxicity (81.3%), but the majority of events were grade 1 (44.6%)^41^. Abemaciclib has unique properties stemming from its preferential targeting of CDK4 over CDK6. This results in less bone marrow toxicity, allowing for continuous dosing. Additionally, abemaciclib has demonstrated superior CNS penetration in comparison to other CDK4/6 inhibitors^42^.

The value of CDK 4/6 inhibitors is not limited to post-menopausal women. The randomized phase II trial, MONALEESA-7, assigned pre-menopausal women to receive either ribociclib or placebo in addition to ET (gonadotropin-releasing hormone agonist and either NSAI or tamoxifen). At 42 months of follow-up, the investigators noted a statistically significant OS benefit with inclusion of ribociclib compared with ET alone (70.2% *vs.* 46.0%; HR 0.71; 95% CI 0.54 to 0.95; *P* = 0.009)^34^. The standard of care for pre-menopausal women without clinical evidence of visceral crisis should include a CDK4/6 inhibitor; evidence-based guidelines support the use of either ribociclib or palbociclib^8^.

OS data are still maturing for palbociclib studies, but MONALEESA-7 and MONARCH-3 have demonstrated superior OS in both pre- and post-menopausal women, respectively^34,43^. Patient reported outcomes for CDK4/6 inhibitors have also shown encouraging results. Quality-of-life studies consistently show that CDK4/6 inhibitors are viewed favorably^44^.

These pivotal trials have led to rapid approvals and expanding indications in MBC in many countries. **Table 1** provides a current overview for administration of these drugs in clinical practice. Overall, the doubling of PFS and encouraging OS benefit with CDK4/6 inhibitors provides unmatched outcomes in first-line treatment. The combination of superior effectiveness and low toxicity profile makes CDK4/6 inhibitors, in addition to ET, the preferred option for patients with hormone receptor-positive, HER2-negative MBC.

**Table 1:** Pivotal trials in treatment of hormone receptor-positive, HER2-negative MBC with CDK4/6 inhibitors

	First-line treatment					Second-line treatment		First- and second-line treatments
	PALOMA-2	MONALEESA-2	MONARCH-3	MONALEESA-7	PARSIFAL	PALOMA-3	MONARCH-2	MONALEESA-3
Design	Phase III, placebo controlled	Phase III, placebo controlled	Phase III, placebo controlled	Phase III, placebo controlled	Phase II, open label	Phase III, placebo controlled	Phase III, placebo controlled	Phase III, placebo controlled
Patients (*n*)	666 post-menopausal	668 post-menopausal	493 post-menopausal	672 pre-menopausal	486 post-menopausal	521 post-menopausal	669 post-menopausal	726 post-menopausal
CDK4/6 Inhibitor	Palbociclib	Ribociclib	Abemaciclib	Ribociclib	Palbociclib (control arm)	Palbociclib	Abemaciclib	Ribociclib
Endocrine partner	Letrozole	Letrozole	Anastrozole or letrozole	Letrozole, anastrozole or tamoxifen + goserelin	Letrozole or fulvestrant	Fulvestrant	Fulvestrant	Fulvestrant
Primary endpoint: PFS (CDK4/6 inhibitor + ET *vs.* ET)								
HR (95% CI; *P* value)	0.58 (0.46–0.72; < 0.0001)	0.56 (0.45–0.72; < 0.00000009)	0.54 (0.41–0.70; 0.00002)	0.55 (0.44–0.69; < 0.0001)	1.13 (0.90–1.5; 0.321)	0.46 (0.36–0.59; < 0.0001)	0.55 (0.45–0.68; < 0.001)	0.59 (0.48–0.73; < 0.001)
Median PFS (months)	24.8 *vs.* 14.5 (Δ10.3)	25.3 *vs.* 16 (Δ9.3)	28 *vs.* 14.7 (Δ13.3)	23.8 *vs.* 13 (Δ10.8)	27.9 *vs.* 32.8 (Δ4.9)	9.5 *vs.* 4.6 (Δ4.9)	16.4 *vs.* 9.3 (Δ7.1)	20.5 *vs.* 12.8 (Δ7.7)
Secondary endpoint: OS (CDK4/6 inhibitor + ET *vs.* ET)								
HR (95% CI; *P* value)				0.71 (0.54–0.95; 0.00973)	1 (0.7–1.5; 0.986)	0.81 (0.64–1.03; 0.09)	0.75 (0.61–0.95; 0.01)	0.72 (0.57–0.92; 0.0045)
Median OS (months)	Not yet reported (August 2023)	Not yet reported (August 2021)	Not yet reported (July 2021)	NR *vs.* 40. 9	4-year OS 67.6% *vs.* 67.5%	34.9 *vs.* 28 (Δ6.9)	46.7 *vs.* 37.3 (Δ9.4)	NR *vs.* 40

HER2, human epidermal growth factor 2; MBC, metastatic breast cancer; CDK4/6, cyclin-dependent kinase 4/6; PFS, progression-free survival; ET, endocrine therapy; HR, hazards ratio; CI, confidence interval; OS, overall survival.

### Use of CDK4/6 inhibitors in subsequent lines of therapy

All 3 CDK4/6 inhibitors have been shown to benefit patients whose disease has progressed on initial therapy with ET without prior CDK4/6 inhibitors. The phase III trial, MONARCH-2, demonstrated the effect of abemaciclib in addition to fulvestrant in patients with previous ET. In this pre-treated population, abemaciclib plus fulvestrant significantly improved median OS to 46.7 months compared with 37.3 months for patients receiving placebo plus fulvestrant alone^33^. Similarly, data from PALOMA-3 supports use of palbociclib in conjunction with fulvestrant in patients for second-line treatment^45^. Fulvestrant plus palbociclib was associated with significant improvement in PFS compared with fulvestrant plus placebo, irrespective of the degree of endocrine resistance, hormone receptor expression level, and PIK3CA mutational status^46^. In the MONALEESA-3 trial, patients were assigned to ribociclib or placebo in addition to fulvestrant in the first or second line^47^. Findings from this trial support the use of ribociclib for patients who have progressed on prior lines of ET without prior CDK4/6 inhibitor exposure.

Notably, abemaciclib is the only CDK4/6 inhibitor with an indication for use as monotherapy. This unique indication was driven by the results of the MONARCH-1 trial which demonstrated abemaciclib’s single-agent activity, even in patients who had progressed through several lines of prior ET^48^.

## Special considerations

### Relapse during or within 1 year of adjuvant ET

Patients who develop recurrent or MBC during or within 1 year of completing adjuvant ET represent a unique challenge in determining therapy. This population may not respond well to ET as first-line setting and is often excluded from trials, including many of the pivotal CDK4/6 inhibitor studies. One option is fulvestrant in combination with palbociclib, per the PALOMA-3 trial^45^. Patients were eligible for this study if they had disease relapse while on or within 12 months of completion of adjuvant ET. This phase III trial showed improved PFS and established this option as an acceptable strategy. Ongoing studies are evaluating other endocrine partners with palbociclib including the PEARL trial^13^. This particular trial assessed the necessity of chemotherapy in patients who developed recurrence while on or within 1 year of an NSAI as adjuvant ET. The study compared capecitabine to exemestane in combination with palbociclib. There was no statistical difference between the 2 regimens, suggesting that chemotherapy is not mandatory in this patient population. Future investigations will continue to assess the appropriate treatment approach for patients with endocrine resistance. An alternative approach is to consider chemotherapy as first-line therapy to achieve maximal response followed by maintenance ET.

### Patients ineligible for CDK4/6 inhibitors

Although the use of CDK4/6 inhibitors for patients with hormone receptor-positive, HER2-negative MBC who are not in visceral crisis is supported by strong evidence, caution should be taken for selected patients to ensure tolerability and safety. Importantly, elderly patients (age > 75 years) appear to have a similar benefit with the combination of CDK4/6 inhibitors and ET, but there is a noticeable increase in toxicity with associated dose reductions and decrease in quality of life. Serious adverse events (grade 3 or 4) occurred in 88.8% of patients > 75 years compared with 73.4% of patients aged < 75 years^49^.

As a general rule, the use of single-agent AI should be considered for selected patients who are unable to take a CDK4/6 inhibitor due to poor performance status or baseline neutropenia or potentially age > 75 years. Single-agent fulvestrant may also be an option for these patients based on the findings of the FALCON trial^20^.

### Concomitant palliative radiation and systemic therapy

Simultaneous treatment with systemic therapy in patients with MBC undergoing palliative radiation raises theoretical concerns about toxicity of combined modality treatment. Although, theoretically, tamoxifen can halt cell proliferation and could inhibit efficacy of radiation, large trials have shown that concurrent tamoxifen does not impair tolerability or outcomes^50^. Similarly, AIs have been safely administered with radiation. The toxicity of concurrent administration of CDK4/6 inhibitors with radiation is unexplored. There is concern for additive toxicity related to neutropenia and fatigue, and murine models demonstrate that palbociclib can be radio-sensitizing and therefore can potentially increase susceptibility to radiotherapy-related toxicity. Retrospective data suggest that CDK4/6 inhibitors may be safely administrated with palliative radiation, but larger studies are needed to confirm this finding before widespread implementation of this strategy^51^. Concurrent administration of other targeted agents including alpelisib and everolimus with radiation has not been studied in breast cancer. Extrapolation from other tumor types suggests safety, but this finding must be validated in larger populations of doses used in breast cancer^52,53^.

### Role of ESR1 mutation as a marker of endocrine resistance

Many mechanisms for endocrine resistance have been proposed including loss of ER expression, altered activity of ER co-regulators, deregulation of apoptosis and cell cycle signaling, hyperactive tyrosine kinase, and stress/cell kinase pathways^54^. Recently, there is much scrutiny targeted toward the ER itself^18^. Specifically, mutations in estrogen receptor 1 (*ESR1*) are believed to play a key role in acquired resistance to ET. It is estimated that 20%–40% of patients with hormone receptor-positive MBC harbor mutations in *ESR1* with higher occurrence in those with more advanced disease^55^. Notably, most of the mutations in *ESR1* develop after adjuvant treatment with AIs, suggesting that this is an acquired resistance to ET.

Presence of these mutations typically portends a poor prognosis. The PADA trial sought to determine the prognostic significance of circulating *ESR1* mutations at baseline in patients treated with AI and CDK4/6 inhibitors^56^. The study confirmed that patients with circulating *ESR1* mutations represent a high-risk population with a worse prognosis (median PFS of 11.0 months) compared with patients who were *ESR-1* wild-type (median PFS of 26.7 months). Interestingly, for patients who cleared *ESR1* mutation after 1 month of treatment, the PFS (24.1 months) was almost equivalent to that of patients who were *ESR-1* wild-type at baseline, suggesting that AI and CDK4/6 inhibitors retain some activity in this population.

## Ongoing studies and upcoming therapies

Given the results of the FALCON study demonstrating superiority of fulvestrant compared with AI in the first-line treatment setting, a recent trial hypothesized that a similar benefit may be seen with combination of fulvestrant with CDK4/6 inhibitors. The PARSIFAL studied the efficacy of fulvestrant coupled with palbociclib, compared with AI combined with palbociclib^57^. The results were presented at the American Society of Clinical Oncology (ASCO) 2020 Annual Meeting and revealed that, at a median follow-up of 32 months, there was no significant difference in PFS or OS between the 2 treatment regimens.

Furthermore, the arrival of immunotherapy in triple-negative MBC prompted investigations in hormone receptor-positive and HER2-postive MBC. Early-phase trials combining immunotherapy with CDK4/6 inhibitors are ongoing, with preliminary results at ASCO 2020 demonstrating safety with this combination^58^.

The success of targeted agents, especially CDK4/6 inhibitors, in MBC has propelled investigators to study these agents in the adjuvant setting. The phase III monarchE trial randomized patients with high-risk hormone receptor-positive, HER2-negative early breast cancer to either ET or abemaciclib in combination with ET in the adjuvant setting^59^. High-risk features included ≥ 4 positive lymph nodes or 1 to 3 lymph nodes and either tumor size > 5 cm, histological grade 3, or Ki-67 > 20%. Very early results demonstrated a significant invasive disease-free survival (IDFS) improvement (2-year IDFS 92.2% *vs.* 88.7%; HR 0.75; 95% CI 0.60–0.93; *P* = 0.01) with addition of abemaciclib to fulvestrant compared with fulvestrant alone. This landmark study raises the possibility of utilizing CDK4/6 inhibitors for high-risk patients to improve outcomes in early breast cancer. Palbociclib was studied in a similar population in the PALLAS trial, but results shared at the European Society of Medical Oncology (ESMO) Virtual Congress 2020 showed that the trial did not meet its primary endpoint of IDFS^60^. Recently, at the 2020 SABCS, the interim analysis findings of the phase III of PENELOPE-B comparing 1 year of palbociclib plus adjuvant ET to placebo plus ET demonstrated that the study did not meet its primary end point of improved invasive disease-free survival^61^. The mixed results from these trials of CDK4/6 inhibitors in early-stage hormone receptor-positive breast cancer suggest that the very promising results seen in MBC may not always translate into benefit in earlier stages of disease. The discrepant findings could also be related to differences in study design/protocol/eligibility or inherent properties of abemaciclib compared with other agents in the class. The NATALEE trial is an ongoing trial evaluating the efficacy of ribociclib in high-risk patients with early breast cancer and may be informative^62^.

## Conclusions

ET remains at the crux of treatment for hormone receptor-positive, HER2-negative MBC. The combination of certain targeted agents with ET has enhanced outcomes for patients. Specifically, CDK4/6 inhibitors have revolutionized the landscape of MBC with doubling in PFS and promising improvement in OS. The efficacy of these agents paired with their tolerability makes them the preferred option for patients with non-visceral crisis hormone receptor-positive, HER2-negative MBC. Special consideration for alternative treatment strategies should be given for elderly patients and those with baseline neutropenia or poor performance status. The ability to perform next-generation sequencing has changed the paradigm of MBC, and routine sampling for PIK3CA mutations is the standard of care. Continued research in the combination of endocrine therapies with novel targeted agents and immunotherapy should continue to bring new hope to patients with hormone receptor-positive, HER2-negative MBC.
